# A heterozygous missense variant in the *YWHAG* gene causing developmental and epileptic encephalopathy 56 in a Chinese family

**DOI:** 10.1186/s12920-022-01377-8

**Published:** 2022-10-15

**Authors:** Zhi Yi, Zhenfeng Song, Jiao Xue, Chengqing Yang, Fei Li, Hua Pan, Xuan Feng, Ying Zhang, Hong Pan

**Affiliations:** 1 Department of Pediatrics, The Affiliated Hospital of Qingdao University, Shandong, China; 2 Department of Central Laboratory, Peking University First Hospital, Beijing, China

**Keywords:** *YWHAG*, Developmental and epileptic encephalopathy 56, Intellectual disability, Early-onset seizures

## Abstract

**Background:**

Developmental and epileptic encephalopathies (DEEs) are a heterogeneous group of severe disorders that are characterized by early-onset, refractory seizures and developmental slowing or regression. Genetic variations are significant causes of these changes. De novo variants in an increasing number of candidate genes have been found to be causal. *The YWHAG* gene is one such gene that has been reported to cause developmental and epileptic encephalopathy 56 (DEE56). Here, we report a heterozygous missense variant, c.170G > A (p.R57H), in the *YWHAG* gene that caused early-onset epilepsy and developmental delay in a Chinese family.

**Methods:**

We described the clinical manifestations of the proband and his mother in detail. Then, we use trio-based whole-exome sequencing to search the etiology of this family.

**Results:**

Both the proband and his mother exhibited early-onset seizures, intellectual disability, and developmental delay. While the proband attained seizure control with sodium valproate, his mother's seizures were not well controlled. Trio-based whole-exome sequencing revealed a heterozygous missense variant, c.170G > A (p.R57H), in the *YWHAG* gene, which was considered as the cause of early-onset epilepsy and developmental delay in this family.

**Conclusions:**

Our report further confirmed that *YWHAG* haploinsufficiency results in developmental and epileptic encephalopathy 56.

## Background

Developmental and epileptic encephalopathies (DEEs) are a heterogeneous group of severe disorders that are characterized by early-onset, refractory seizures and developmental slowing or regression. Genetic variations are significant causes of DEEs [1, 2]. With the wide application of whole-genome and whole-exome sequencing, an increasing number of candidate genes have been found to be causal, but most of these are discovered in only a small proportion of cases [3]. *YWHAG* (* 605356) is one such gene that has been recently related to DEEs.

The *YWHAG* gene, located on 7q11.23, which encodes YWHAG, is a member of the 14-3-3 protein family and is highly expressed in the brain, skeletal muscle, and heart [4]. Eleven variants in the *YWHAG* gene have been reported to cause developmental and epileptic encephalopathy 56 (DEE56) (# 617665) or autism [3, 5–8]. The affected patients exhibit early-onset epilepsy, intellectual disability, motor developmental delay, speech impairment, and sometimes behavioral problems [3, 5]. However, the disorder has great clinical heterogeneity, as some patients may have a mild phenotype, with normal delivery and normal development, and only epilepsy is observed [9]. Here, we report a missense variant c.170G > A (p.R57H) in exon 2 of *YWHAG,* which caused early-onset epilepsy and developmental delay in a Chinese family.

## Methods

### Ethical approval and participants

Written informed consent for participation and whole-exome sequencing and subsequent Sanger sequencing as a part of the diagnostic process (approved by the Medical Ethical Committee of Affiliated Hospital of Qingdao University) was obtained from the proband’s parents and grandparents. Detailed clinical manifestations from the proband and his mother were then collected.

### Whole-exome sequencing

To explore the etiology of the early-onset seizures and developmental delay in this family, we performed trio-based whole-exome sequencing on the proband and his parents. Peripheral blood samples (2 ml) were collected from the boy and his family members. Then, the samples were sent to MyGenostics, Beijing, for trio-whole exon sequencing using the Illumina HiSeq X Ten system. Following sequencing, raw image files were processed using Bcl2Fastq software (Bcl2Fastq 2.18.0.12, Illumina, Inc.) for base calling and raw data generation. Then the clean reads were aligned to the reference human genome (hg19). The final identified variants were evaluated using three algorithms, PolyPhen-2 (http://genetics.bwh.harvard.edu/pph2/), Sorting Intolerant From Tolerant [SIFT; (http://sift.jcvi.org/)], and MutationTaster (http://www.mutationtaster.org/), to predict pathogenicity. Variant interpretation was performed according to the American College of Medical Genetics (ACMG) guidelines [10]. Sanger sequencing was utilized for further validation of variants and variant detection in other relatives.

## Results

### The proband

**Fig. 1:**
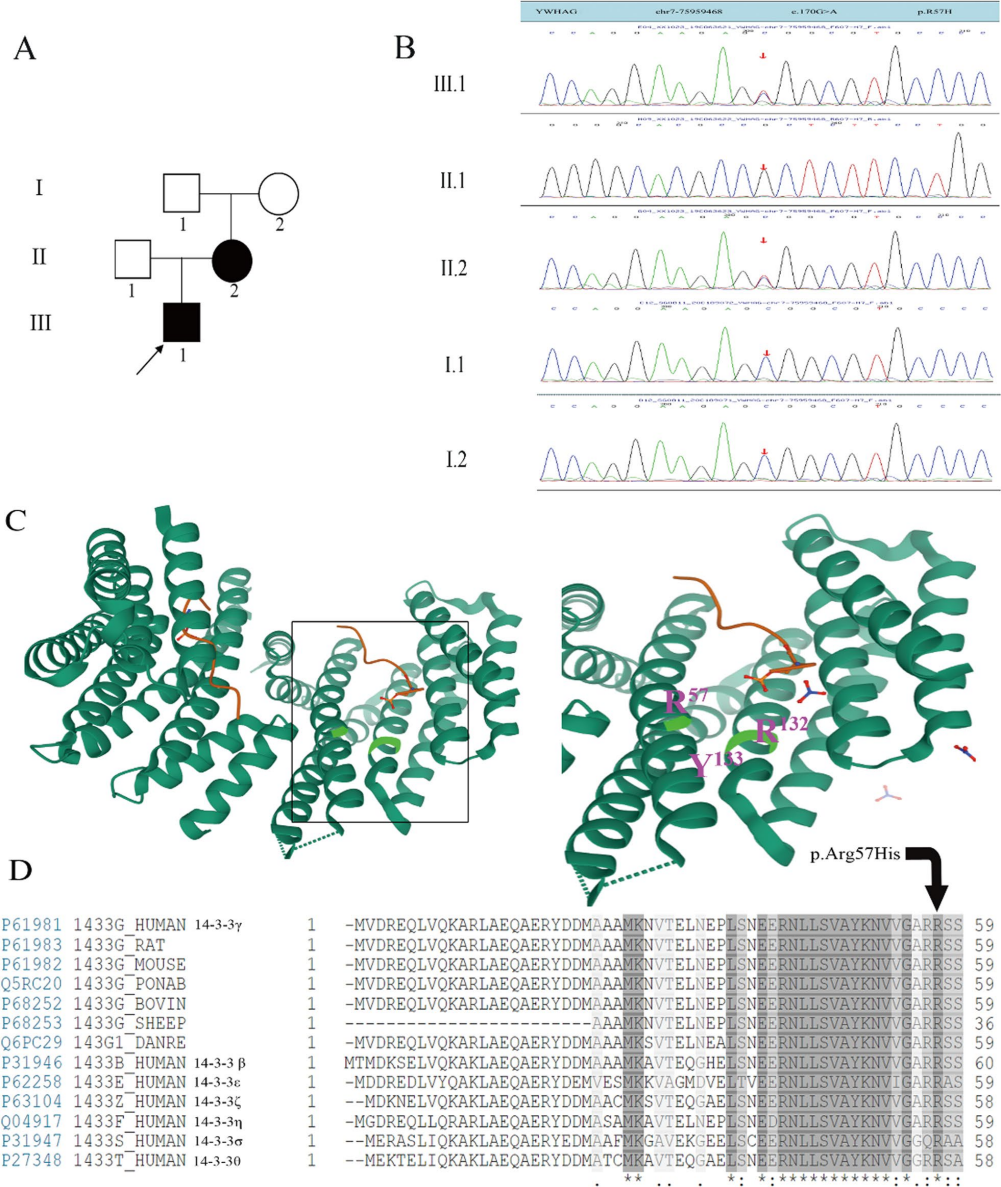
**A**: The family genogram, in which the black arrow indicates the proband. The proband is represented by a black box, and the affected mother is represented by a black circle. **B**: Sanger sequencing results of the family. The red arrow points to the variant site. The proband and his mother carry the variant, but the father (II.1), the grandfather (I.1) and the grandmother (II.2) do not carry the variant c.170G > A (p.R57H). **C**: crystal structure of YWHAG (PDB:3UZD). Left: Dimeric YWHAG is shown as bottle green ribbons, and the phosphopeptide ligand is shown as an orange stick. Right: close-up view of the binding groove and side chains of the residues crucial for phosphopeptide binding. The conserved triad of two arginines and a tyrosine residue (Arg-57, Arg-132, and Tyr-133), which form the positively charged patch, are shown in green. **D**: Partial sequence alignment of YWHAG orthologs and different human 14-3-3 proteins surrounding the variant. Identical residues across all proteins are shown in black

The patient (III.1, Fig. 1 A) is a boy who was born at full-term via cesarean section. His parents are nonconsanguineous. His father is healthy. His mother (II.2, Fig. 1 A) has seizures and developmental delay. He was the first child of his parents, and his mother had no history of miscarriages. His birth weight was 3200 g (p50), birth length was 50 cm (p50), and birth head circumference was 34 cm (p50). There was no history of asphyxia and anoxia. He has no unusual features. His gross motor development has been delayed since birth. He could raise his head at the age of 4 months, turn over at the age of 6 months, sit at the age of 8 months and walk without assistance at the age of 1 year and 6 months. He is now 3 years and 10 months and cannot jump. His language development is seriously delayed. Now, he can say only “papa, mama, no” or express his needs by pointing to the object. He can understand simple commands. He is very irritable. He had his first seizure at the age of 1 year and 11 months, manifested as upward gaze of the eyes, lip cyanosis, and limb trembling, which is considered a generalized tonic–clonic seizure (GTCS). EEG at that time did not reveal a seizure and showed no abnormalities in the background. Neuroimaging was unremarkable. Sodium valproate treatment was initiated, and he was seizure free after 4 months of treatment. Interval EEG and brain MRI performed again at 3 years and 10 months showed no abnormalities.

### The proband’s mother

The proband’s mother (II.2, Fig. 1A) had her first seizure at the age of 2 years, also manifested as GTCS. Her relatives did not report other types of seizures. Interval EEG was unremarkable. She was treated with antiepileptic drugs, but it is not known what they were, and she had a 5- to 6-year remission of epilepsy. During pregnancy, she stopped taking drugs without the guidance of a doctor, and the seizures returned. She is currently being treated with carbamazepine, phenytoin sodium and sodium valproate, but the seizures are not completely controlled. The mother also had global developmental delay. She sat at the age of 8 months and walked without support at the age of 1 year and 5 months. She did not do well in school. She can cook, but she cannot shop. At examination, she could understand some things but could not express herself very well. She refused to take intelligence tests and refused further examination with EEG and brain MRI.

### Whole-exome sequencing results

We performed trio-based whole-exome sequencing on the proband and his parents. We identified a heterozygous variant c.170G > A (p.R57H) in exon 2 of *YWHAG* (NM_012479), which was inherited from his mother (Fig. 1B). Further investigation with Sanger sequencing found that the grandmother and grandfather did not carry the variant (Fig. 1B). This is a heterozygous variant that causes the arginine at position 57 to be replaced by histidine. PolyPhen-2 predicted this variant to be damaging, with a score of 1.000. The variant was also predicted to be disease causing by MutationTaster and damaging by SIFT (with a score of 0.000). According to ACMG guideline, the p.R57H variant in *YWHAG* gene meets the criteria to be classified as likely pathogenic (PS2 + PM2 + PP1 + PP3 + PP4) [10]. This site is highly conserved between species and subtypes of the protein family, and the arginine at position 57 is part of the highly conserved triad of two arginines and a tyrosine (Arg132-Arg57-Tyr133) that normally form a positively charged patch within a binding groove for interacting phosphopeptides [3, 11]. This ability of the protein to bind phosphopeptides is potentially affected by substitution at this site (Fig. 1C and D).

## Discussion

### Functional study of YWHAG

The *YWHAG* gene is located on 7q11.23, which encodes YWHAG, a member of the 14-3-3 protein family and is highly expressed in the brain, skeletal muscle, and heart [4]. 14-3-3 proteins function in vital cellular processes, such as metabolism, protein trafficking, signal transduction, apoptosis and cell cycle regulation [12]. 14-3-3 proteins exist in monomeric and dimeric states as homo- and heterodimers, respectively, but YWHAG is almost entirely dimeric. Each monomer consists of a bundle of nine α-helices (αA to αI), of which helices αC, αE, αG, and αI form a conserved peptide-binding groove, which has a positively charged patch on one side and a hydrophobic patch on the other (Fig. 1 C). The positively charged patch is formed by a conserved triad of two arginines and a tyrosine residue (Arg-57, Arg-132, and Tyr-133), which bind the phosphate group of the interacting phosphopeptide/protein [3, 11, 13]. In previous study, de novo variants in the *YWHAG* gene were predicted to impair dimerization (p.E15A) and phosphopeptide binding (p.R132C)[3]. In the same way, the replacement of arginine by histidine at position 57 was predicted to affect the ability of the protein to bind phosphopeptides, thus affecting the biological function downstream. Previous functional experiments carried out by knocking down *ywhag1* in zebrafish revealed reduced brain size and increased diameter of the heart tube in zebrafish [14]*.* Other researchers found that both the overexpression and the knockdown of *Ywhag* in mice disrupt neuronal migration of pyramidal neurons, which indicates that a balance of Ywhag expression is required during cortical development to prevent delays in neuronal migration [15, 16]. Kim et al. found that *Ywhag* homozygous knockout mice was prenatally lethal, and heterozygous mice showed developmental delay. In addition, behavioral analyses found that heterozygous mice display hyperactive and depressive-like behavior along with more sensitive responses to acute stress than littermate control mice [17]. In summary, we hypothesize that the variant affecting the ability of the protein to bind phosphopeptides may also result in delays in neuronal migration, thus leading to developmental delay, epilepsy and so forth.

### Clinical manifestations and diagnosis

Until now, fourteen variants in 25 patients were found to result in *YWHAG* deficiency (including our patients) [3, 5–8, 18–20]. The cases of fourteen of the patients were described with detailed clinical manifestations (Table 1). Affected patients mainly manifested as early-onset epilepsy, mild-moderate intellectual disability, motor developmental delay, speech impairment, and sometimes behavioral problems. Some of them have facial dysmorphism such as prominent forehead, long palpebral fissures, bulbous nose, and absent Cupid's bow. However, a mild phenotype was also observed, as one patient was reported to have normal development, and only epilepsy was observed[9]. This suggests the great clinical heterogeneity of this disease. The age of seizure onset is usually less than 2 years. The most common seizure types are GTCS, absence seizures and myoclonic seizures. One of the patients reported by Kanani et al. experienced only a single generalized tonic–clonic seizure, although most of the patients had multiple seizure types and multiple seizures. Most of the patients had normal interictal EEG and cranial MRI results (Table 1) [3, 5]. Both of our patients had mild-moderate ID, motor developmental delay, and speech impairment. They experienced multiple GTCSs but no other types of seizures. The proband was irritable. Although quite a few patients had facial dysmorphism, no facial dysmorphism were observed in our patients. A recent paper reported a patient carrying the same variant as our patient, whose clinical presentation was roughly the same as ours. But, some of his EEG recordings showed generalized or bifrontal spikes and SW complexes [19]. A total of fourteen variants have been found so far. Except for the variants shown in Table 1, a de novo p.D129E variant was reported in an individual with Lennox-Gastaut syndrome (LGS) [6]. A p.K50Q de novo variant was identified in a subject with autism [7]. Very recently, two novel variants (p.R42Ter and p.K125E) were reported in two unrelated families with childhood myoclonic epilepsy and FS. They all have normal intelligent and motor development [20]. And another variant p. R132H was reported in a patient with DEE56 in a cohort study [18].Among these patients, there seem to be hot spot variants. p.R132C was found in 4/15 patients, and p.Y133S was found in 2/15 patients (Table 1). In addition to the patient whose case is summarized in Table 1, the p.Y133S variant was reported in a patient severely affected by a neurodevelopmental disorder who had no detailed clinical description[8]. If a patient has a phenotype similar to that described above, the diagnosis can be established by genetic analysis to identify a pathogenic variant in the *YWHAG* gene.

**Table 1:** The variants and clinical manifestation of reported patients (only those patients with detailed clinical descriptions)

	Literature [3]				Literature [5]						
	Subject B	Subject D	Subject E*	Subject F	Patient 1	Patient 2	Patient 3	Patient 4*	Patient 5	Patient 6	Patient 7
Variant	c.394C > T (p.R132C)	c.44A > C (p.E15A)	c.394C > T (p.R132C)	c.394C > T (p.R132C)	c.169C > G (p.R57G)	c.398A > C (p.Y133S)	c.532A > G (p.N178D)	c.394C > T (p.R132C)	c.394C > T (p.R132C)	c.169C > T (p.R57C)	c.529C > A (p.L177I)
Inheritance	De novo	De novo	De novo	De novo	De novo	De novo	De novo	De novo	De novo	De novo	De novo
Sex	Female	Female	Female	Female	Female	Male	Male	Female	Male	Female	Female
Age	18 years	10 years	22 years	15 years	15 years	16 years	7 years	23 years	4 years	10 years	7.5 years
ID	Mild-moderate	yes (IQ ca.55)	yes (moderate to severe)	WPPSI III (6 years), VIQ 73, PIQ 58	Mild–moderate	Mild–moderate	Moderate	Moderate	Mild	Moderate	Mild–moderate
Speech	Early language delay	Delayed	Delayed	Mildly delayed	Normal	Normal	Speech delay, echolalia	Delayed	Mild delay	Requires support. Unclear speech. Short simple sentences	Delayed
Walking	Unknown	Unknown	Unknown	Unknown	15 m	23 m	24–30 m	16 m	21 m	22 m	18 m
ASD	ADHD	–	–	–	present	–	present	present	–	–	–
Age of seizure onset	12 months	6 months	< 6 months	< 6 years (unknown)	10 months	16 years	2 years	< 6 months	2 years	< 5 years	–
Seizure type	Generalized myoclonic, atypical absence, generalized tonic–clonic	Prolonged seizure with fever and then two episodes of status epilepticus associated with regression and hemiparesis	Myoclonic, prolonged generalized tonic–clonic with fever, generalized myoclonic, absence, generalized tonic–clonic	Absence, eyelid myoclonia, myoclonic, persistence of absence seizures	Absence, focal and generalized tonic–clonic	Isolated generalized tonic–clonic	Absence	Generalized tonic–clonic, generalized myoclonic, absence	Generalized tonic–clonic	Frontal lobe epilepsy	Absence
Anti-epileptic drugs	Clonazepam, lamotrigine, divalproex sodium, ethosuximide	Divalproex sodium	Divalproex sodium, stiripentol	Divalproex sodium, lamotrigine	Levetiracetam, ethosuximide	None	Ethosuximide	Stiripentol, divalproex sodium	Sodium valproate	Sodium valproate, carbamazepine	None
Treatment resistant?	No	No	Partial response	No	No	N/a	No response to LTG	Partial response	No	No	N/a
Ictal EEG	Myoclonic jerks, generalized spike-and wave discharge	Not available	Spike-and-slow wave, poly-spike, and slow-wave discharges	Not available	–	Not performed	–		–	–	–
Interictal EEG	2 years: dysrhythmic background, generalized atypical spike wave, frequent bifrontal spikes; 14 years: dysrhythmic background, rare sharp waves in bianterior quadrants	Not available	21 months: generalized 3 Hz spike wave with absence seizures; 10 years: generalized polyspike wave with myoclonic seizures; 14 years: occasional spike wave	8 years: bilateral frontotemporal spikes, generalized spike waves	–	Not performed	Prolonged burst of generalized 2.5 Hz spike and wave activity noted	Generalized polyspike wave and slow wave discharges	–	–	Normal
Cranial MRI	3 years: asymmetric brainstem not thought to be significant	Generalized atrophy with diffuse loss of white matter	10 years: normal	Normal	Normal	Normal	Normal	Normal	Focus of hyperintensity frontal lobe	Subtle signal changes in frontal subcortical white matter	Normal

	Literature [9]	Literature [19]	Our patients	
			Proband	Mother
Variant	c.619G > A (p.E207K)	c.170G > A (p.R57H)	c.170G > A (p.R57H)	c.170G > A (p.R57H)
Inheritance	De novo	De novo	Inherited	De novo
Sex	Male	Male	Male	Female
Age	4 years 9 months	8 years	3 years 10 months	34 years
ID	Normal	Mild	Moderate	Mild–moderate
Speech	Normal	Delayed	Delayed	Delayed
Walking	16 m	15 m	18 m	Unknown
ASD	–	ADHD	–	–
Age of seizure onset	9 months	1 years 6 months	1 years 11 months	2 years
Seizure type	Myoclonic jerks with extension of the hands with no clusters of spasms(9 months). Short events of staring with eye blinking. Head and eye deviation to the left and staring (3 years10 months)	Absence seizures, febrile and afebrile generalized tonic–clonic seizures	Generalized tonic–clonic	Generalized tonic–clonic
Anti-epileptic drugs	Valproic acid, levetiracetam	valproic acid	Sodium valproate	Phenytoin sodium, carbamazepine, sodium valproate
Treatment resistant?	Partial response to valproic acid, seizure free with levetiracetam	No	No	Partial response
Ictal EEG	Generalized spike waves with bilateral frontal predominance(9 months)	–	–	–
Interictal EEG	Generalized discharges of spikes with no decrement and no hypsarrhythmia pattern(9 months) Spikes at the right posterior frontal region and independent on the left posterior frontal region(3 years 10 months)	Used to be normal or mildly abnormal to background slowing. Only some EEG recordings showed generalized or bifrontal spikes and SW complexes	Normal	Normal
Cranial MRI	Some nonspecific hyperintensity signals on fluid-attenuated inversion recovery (FLAIR) sequence	Normal	Normal	–

^*^Subject E reported by Guella et al. and patient 4 reported by Kanani et al. was the same patient

### Treatment and prognosis

The seizures of most patients are sensitive to antiepileptic drugs. Usually, seizure control can be achieved by sodium valproate and levetiracetam and ethosuximide and stiripentol. One patient was found to have no response to lamotrigine [5]. The proband in our study attained seizure control after 4 months of treatment with sodium valproate. However, his mother attained only partial control with phenytoin sodium, carbamazepine, and sodium valproate. This may be partly due to the sudden withdrawal of drugs during pregnancy and the subsequent irregular use of drugs after pregnancy. Motor and speech rehabilitation may be useful to patients; however, due to the small number of cases, there are no data to support this. In light of the reported patients and our patients, most patients with DEE56 seem to have a relatively good prognosis. They may achieve self-care ability, and the seizures can be controlled with standard antiepileptic drugs [5].

## Conclusion

*YWHAG* haploinsufficiency is now a recognized etiology of DEE56. From the reported patients and our cases, we can see that although patients with DEE56 have early-onset seizures and global developmental delay, most of them seem to have a relatively good prognosis. We report a missense variant in the *YWHAG* gene in a Chinese family, which will expand the variant spectrum and further enrich the genotype–phenotype relationships of DEE56. Since some patients may get married and have children due to the relatively good prognosis, we also emphasize the importance of genetic diagnosis and prenatal diagnosis for females who have mild-moderate intellectual disability, developmental delay and especially epilepsy.

## Data Availability

The variant is available in the ClinVar repository [https://www.ncbi.nlm.nih.gov/clinvar/]. the accession number is SCV002064257. The raw sequence data reported in this paper have been deposited in the Genome Sequence Archive[21] in National Genomics Data Center [22], China National Center for Bioinformation / Beijing Institute of Genomics, Chinese Academy of Sciences (GSA for human: HRA002140) that are publicly accessible at https://ngdc.cncb.ac.cn/gsa-human/. Please refer to the following website for details https://ngdc.cncb.ac.cn/gsa-human/browse/HRA002140.

## Abbreviations

DEE: Developmental and epileptic encephalopathies
GTCS: Generalized tonic-clonic seizure
LGS: Lennox-Gastaut syndrome
ACMG: American college of medical genetics
